# A Lecture on Some Points in the Clinical History and Pathogeny of Locomotor Ataxy

**Published:** 1870-03

**Authors:** Meredith Clymer


### A LECTURE ON SOME POINTS IN THE CLINICAL HISTORY AND PATHOGENY OF LOCOMOTOR ATAXY.

BY MEREDITH CLYMER, M.D.

Read before the Medical Society of the County of New York, at the Meeting held January 3, 1870.

During the past few years, a handful of workers have investigated with zeal, care, and intelligence, the group of clinical facts familiar to us by the name of locomotor ataxy. Still there is much conflict of opinion concerning its nature, and many of the theoretical imaginings, though ingenious, are unsatisfying, and would seem to need modification or correction. The invitation of the President of the Society gives me the privilege of offering for your consideration some points in the clinical history and pathogeny of this curious disorder. The purpose of these fragmentary remarks is to get an expression of views on these difficult and unsolved problems, and to invite a discussion which may assist towards their right understanding.

The name locomotor ataxy is not a happy one; it sets forth a single objective symptom—motorial inco-ordination—which is not prominent, even if present, in the early stages, and is not proper to this disease, for it is met with in many other and very various affections. In these instances, however, muscular ataxy is an occasional and not an inevitable sign, often quite transitory, and joined with other phenomena, each series unlike the other, and agreeing respectively with the particular intrinsic lesion. But in the disease we are considering, it necessarily appears, sooner or later, in its course, and is constantly linked with certain morbid expressions having a regular sequence and pathogenetic interdependence, and is, moreover, always related to the same material cause. Few maladies have a more emphatic natural history than locomotor ataxy, or a better right to be recognized as a distinct pathological species.

Let me briefly define motorial ataxy or asynergia: A centric nervous disease, manifested by certain sensory and motory disorders, as, sight troubles, transitory or permanent; sudden violent darting, or tearing, or burning, or boring pains, most often in the lower limbs, with other perversions of sensation; impaired adjustment in the several groups of muscles which naturally work harmoniously together in such acts as standing, walking, and handling; muscular power often but slightly lessened; sexual appetite and ability frequently impaired or abolished; nearly always progressive in its course; the anatomical characters being sclerosis of the posterior columns, disintegration of the gray substance of the posterior cornua, of the spinal cord, with atrophy of the posterior nerve-roots, and sometimes structural change in the cranial nerves, chiefly the second, third, and sixth.

Usually an ataxic seeks medical advice in one of two conditions: (1.) He finds that he gets easily tired after walking, standing, or going up stairs; the fatigue is more rapid and greater from standing than from walking, for many who can still get over a good deal of ground comfortably cannot remain standing for a few minutes, the sense of weariness being so intolerable. Locomotion may be yet natural in its harmony and succession, whilst the attention is fixed on it; but it is no longer merely a mechanical act; unless the limbs are watched the movements lack their accustomed precision, and are awkward and blundering. Walking, when once begun, commonly ceases to be a cerebral act; but here its continuance requires incessant volitional mediation, cerebral innervation coming in to supplement diminished spinal innervation. The patient, too, will probably complain of not being sure of his legs, of his feet feeling at times as if weighted, of their not "taking hold of the ground," of frequent stumbling, of losing balance on turning round quickly, of some stiffness about his joints, and of difficulty in starting off in his walk. Bid him shut his eyes, and stand with his feet brought together, and he will probably totter; or make him walk with the eyes closed, and he may reel and stagger, or even topple over. At this time the gait is generally peculiar—the steps short and quick, the heel striking the ground first; the characteristic hitchy, catching-up, stamping gait generally comes later, with plantar anæsthesia, giving the feeling as if the soles of the shoes were of wood or sponge, or that the individual was walking through sand, or loose grain, or straw. (2.) In the other instance, the unsteadiness of gait has been first noticed towards evening, or in the dark: there is consciousness of a want of proper control of the legs; the way is felt with hesitation, in perpetual fear of falling over, and there is unwillingness to go about even in familiar places without a light.

Now, in these cases there will be most likely a history of ocular troubles, as weakness of sight, defective visual accommodation, or strabismus, or ptosis, or double vision; they may be still present, more or less persistent from their outset, or occasional and recurrent, or may have passed away. Rheumatic aches and neuralgic pains, flying or fixed, will almost certainly have been felt. These pains are very significant; mobile and intermitting, they shoot like lightning or an electric shock through a limb, or the face, or scalp, or some part of the trunk, or genital organs; or they may be only or chiefly in one limb, flashing upwards through it, or localized in some part of it, as the sole of the foot, in which case they are boring or gnawing, or pressing, coming on in spells, lasting for hours or even days, and recurring weekly or monthly. They are described sometimes by patients as the toothache in the legs. Sometimes they are fixed, very circumscribed, constant, and agonizing, and hinder sleep (tabes dolorosa of Remak).

The sight-troubles and the limb pains are rarely though at this time connected by the patient with his limb-weariness or his unsteady gait, particularly in the dark; these latter occupy his mind, and make him uneasy from mistrust of threatening palsy.

My own observations have led me to believe that there is a *formative stage* of this disease, betokened by certain symptoms, and which, so far as I know, has not been noticed by writers. They happen some time before the usual initial ocular disturbances, or the pain-spells, or unequivocal unsteadiness of gait, or indeed any of the symptoms usually attributed to the outset of the malady, and are mostly shown in the upper segments of the body. They consist of slight motory deficiences and sensory annoyances, both often so insignificant as to escape the attention of their subject or his intimates. There will be awkwardness in handling small objects, as picking up a pin, or match, or putting a lead in a pencil, tying a knot, fastening a cravat; the fingers are clumsy and not alert; things are not grasped, held, and taken up with wonted nimbleness and dexterity; they are no sooner seized and lifted than they are dropped; unpleasant sensations are felt in the skin of the face, as the tip of the nose, small areas in the temples, cheeks, lips, and are spoken of as tinglings, crawlings, as if an insect had alighted upon or was moving over the part; there may be occasional deep-seated quiverings of the flesh, particularly in the eyelids, or lips, or cheek;* a feeling of numbness, confined to a small space in one of the lips, or of being suddenly touched there by something very hot or very cold, with a tendency to fall asleep in the arms and legs, when sitting or lying down, giving the sensation of "pins and needles," particularly in the fingers and toes. Some indecision in the motions of both the upper and lower extremities may exist, with occasionally a notion of their being weighted, stiffness of the joints, effort in rising from a seat, weariness after small muscular exertions, with steady aching in the nape of the neck and small of the back, and lessened sexual aptitude. Neuralgia may be occasional, but the ocular disturbances are either wanting or only manifest in the form of defective visual accommodation, or con-

*Since this paper was read, the writer has been consulted by an ataxic in whom the first symptom noticed was an unpleasant feeling of formication, numbness, and twitchings in small areas of the temples. His physician told him that it came "from his stomach, general running down of his system, and smoking cigars." Three months afterwards he "began to lose his hold of the ground."

tracted pupils. If experience shall confirm the constancy of an inceptive stage with certain signs, we may perhaps be able to meet this insiduous disease at a period before serious structural disorganization has taken place, and successfully check its subtle advances.

The *integrity of muscular power* has been insisted on by Duchenne, C. B. Radcliffe, and others, and held to be a diacritic feature. I believe with Oppolzer, Brown-Séquard, Lockhart Clarke, Benedikt, and Allbutt, that it is always more or less impaired, and that, too, from the beginning. Close attention will show some degree of muscular weakness as one of the earliest phenomena, but in such small degree as to be overlooked unless carefully inquired for. It is true that in very many cases of the developed disease the patient, when lying or sitting, can execute the ordinary limb-motions with promptness and energy; he may lift even heavy weights attached to his feet, and will resist with much apparent show of strength all attempts to bend or straighten his limbs against his will by another; but here, as in walking, extraordinary cerebral interference comes to the aid of lessened spinal ability, and observers have been misled by overlooking this circumstance, as well as by faulty modes of investigation. The real state of motor force in the limbs is properly measured by the degree of perfectness of their quasi-automatic functions, and this is palpably damaged from the outset. The duration of each act of contraction on the part of any muscle, or group of muscles, interested in the given movement is perceptibly shortened, and this accounts for the constant weariness and easily induced fatigue after moderate exercise; and from the necessary lengthened cerebral action results the great exhaustion after any effort demanding large or sustained expenditure of muscular force. Long walks or fitful feats of strength are possible, but these are extraordinary volitional acts, tax severely the brain, are followed by great nerve depression, and are no proper test of the actual amount of muscular power. Another source of error has lately been mentioned by Dr. Brown-Séquard. He writes: "In ataxics, from lesions of the posterior columns of the cord, involuntary spasmodic movements are often mixed with those purely volitional, thus giving to these the appearance of as much, or even more power, as in the natural state; from which follows the assertion that there is no palsy, whilst there really is, and that too often in a notable degree." (Annales de Physiologie, Nor. et Path., t. ii., p. 761, 1869.)

Dr. Jaccoud insists that when the ataxy is well marked, reflex motility will, as a rule, be found heightened, and that this excess lasts to an advanced stage. This, as a general statement, will, I think, be found incorrect, and that in most cases reflex action is really weakened. But now and then we see instances where it is terribly increased. Let two ataxics be requested to lie down, and each one to put out a leg. In one the act will be done naturally, or nearly so; the limb is practically controlled by the will; the movement stops at the time desired; while the leg of the other will be thrown out, probably striking a bystander, and begin to jerk and jump about, with no ability on the part of the patient to check its extravagance.* In other cases, like saltatory spells may be brought on by rubbing or tickling the soles of the feet, or by applying cold to the limb, or sometimes by only uncovering it, and occasionally they come on without seeming provocation. They are generally attended with anxiety, and back and limb-pains. Jerkings of the lower extremities, when about falling asleep, are often noticed by ataxics, and one of my patients complained of having had several times general convulsive movements, producing the sensation as if he were suddenly tossed up from a spring-board.

It was first mentioned by Jaccoud, and about the same time by Benedikt, that many ataxics, whilst standing, are subject to involuntary intermittent muscular contractions, particularly of the flexors of the foot, the tendons becoming very prominent on the dorsum, and sometimes so vigorous as to lift the foot from the ground on the heel. This, and the fibrillary twitchings already alluded to, may be referred to the same class of reflex phenomena.

In a large majority of cases there is a gradual *decay of sexual feeling* in both sexes, and which goes on to total extinction. But there are some remarkable instances of extraordinary exaltation both of desire and capacity in the first stage of the disease. Trosseau has related some of these exceptional examples in the male, and Dr. Bouchard tells of a woman in whom, at the outset of the affection, sexual desire became so imperious as to require frequent satisfaction during the day as well as the night; and this state was followed by complete anaphrodisia, coition causing no special sensation. In males, and coincident with loss of desire, or impotency, or hasty sem-

* In these phenomena we have what Dr. Brown-Séquard has called *spinal epilepsy*, which includes those cases of spasms, clonic or tonic, of the lower limbs, which are pathogenetically in direct relation to some lesion of the spinal cord causing exaggerated reflex excitability.

inal discharges, nocturnal emissions are common, and females are liable to dreams with voluptuous sensations. In these conditions I have always found in the male a marked fall in the temperature of the genital organs, and often to a degree readily appreciable by the hand.

The *pulse-rate* is almost constantly quickened in ataxics, and it is important to bear this in mind when deciding on the nature of any intercurrent disorder. In eight cases Dr. Charcot found the mean pulse-rate to range from 90 to 100, and in some it has between 100 to 124. There is no corresponding increase in the body-heat. Eulenberg's sphygmographic observations show considerable lessening of arterial tension.

I will now briefly mention some epiphenomena which happen more or less often in the course of locomotor ataxy, and which have been studied only quite recently. They have all peculiar traits, and seem to have a direct pathogenetic relation to the disorder.

The *stomach troubles* have been generally overlooked by writers; they have special characters, and are not infrequently intercurrent in the developed disease, though sometimes met with at the outset. These gastric attacks begin with sense of weight and darting pains in the epigastrium, with general abdominal fulness. In a day or so, sometimes sooner, there are hiccup and nausea, followed by repeated retchings and vomitings, the matters thrown up being food, bile, acid, etc. During these spells, the limb-pains are worse, and sharp pains dart from the pubes towards the stomach.

*Heart troubles* may accompany these gastric paroxysms; they consist of sudden and violent palpitations, with painful spasms and a feeling of constriction in the cardiac region. They commonly last but a short time, and during their continuance no *bruit de souffle* or other physical sign is heard.

Very lately Dr. Fereol has published some interesting observations of certain *laryngo-bronchial symptoms* to which ataxics are liable. They will be seized without warning with a spasmodic cough, threatening suffocation, lasting for a few minutes, and then suddenly ceasing, and neither accompanied nor followed by expectoration. I have long remarked that when an ataxic has an ordinary cold, his cough-spells are very distressing, convulsive, and obstinate, with a whistling through the larynx, and sometimes crowing back-draughts.

Now, in these several organic disturbances the pathogenetic predominance of the nervous element is apparent, and the interesting question naturally suggests itself, what part the sympathetic plays in their production and mechanism.

Drs. Charcot and Ball, of Paris, have lately investigated the symptoms and nature of the curious *joint affection* which happens in the beginning of the second stage of the disease, or during the interval of the first and second stages, and which hitherto had been regarded as a rheumatic and accidental complication. They have attempted, however, to show that it has no kinship with rheumatism, and is directly connected with the original disease. It occurs without any premonitory indications, the limb suddenly swelling, the tumefaction being more marked about the implicated joint. There is neither redness nor pain, nor constitutional disturbance,* nor increased body-heat. The general puffiness is not like ordinary œdema, for there is no pitting on pressure, but, on the contrary, rather resistance. The effusion into the articulation (hydrarthrosis) is large and constant. The knee-joint would seem to be the site of election, with nearly always marked unilateral predominance. In the eighteen observations collected by Dr. Ball the ankle-joint was not once involved, though in Case 2 it is stated that the patient had once sprained his ankle and had never been able to use it perfectly since, with the remark that though at the time it had been regarded as an accident resulting from weakness of the limb, it might have been perhaps a primary manifestation of spinal arthropathy. In one of my ataxics hydrarthrosis of the ankle-joint preceded immediately the outbreak of the motorial troubles. The patient had had severe facial neuralgia and lessened sexual aptitude for some time, when his ankle-joint suddenly became swollen, without redness or pain; this hindered walking, and on applying to his physician he was told that it was a sprain, and treated accordingly; the local symptoms soon went away, but immediately afterwards he had a cottony feeling in the soles of his feet, and his gait became unstable.

The duration of the joint-affection is uncertain; it may disappear after a few weeks, leaving no ill effects; or it may last for months; and sometimes the morbid processus is indefinitely prolonged, leaving permanent deformities. When recovery is apparently rapid, there is always great risk of a relapse. The resulting changes in the end of the bones forming the joint have been described by the opposing terms hypertrophy and atrophy; and this double pathological condition is especially well marked in the knee-joint. Ossific tumors are developed, while the articular surfaces seem to undergo rapid destruction. In the only case in which there was an autopsy, the cartilages had disappeared, the bony substance itself was, as it were, eaten

* There was one exception in the eighteen cases collected by Ball.

away, and small osseous excrescences surrounded the joint. As a result of the atrophy of the articular surfaces, irreducible dislocations are often noticed. The effused fluid is not confined to the synovial sac, but extends to the neighboring bursæ, it is in large quantity, citron-colored, serous, and contains neither pus, nor blood, nor albuminous flocculi. Well-marked anatomical differences separate this form of arthropathy from certain other articular affections which have some resemblance to it, but which are in fact very distinct. In spinal arthropathy the peculiar vegetations and phosphatic incrustations met with in chronic arthritis are absent, as well as the augmentation and flattening of the articular surfaces. In the joint-affections following paraplegia, we have the effects of prolonged immobility; the structure of the bones is modified, and the polish of the surfaces is wanting, but the peculiar atrophy is not seen. It has a certain resemblance to the dry caries of Volkmann: for in this, too, there is rapid atrophy of the articular surfaces; but the serious changes in the bony tissue itself, producing the honey-combed cavities lined by a firmly adherent, delicate granulated membrane, which is the anatomical character of *caries sicca*, is not seen in spinal arthropathy. It was, as I have said, for a long while looked on as of rheumatic origin and constitution; but the absence of local pain and redness, and of fever, with natural body temperature, would alone seem to be conclusive of the non-identity of the two affections. Rheumatism, too, is disposed to be migratory, and may invade several articulations successively, whilst in ataxic arthropathy there is a tendency to localization in one joint, or at most in two. When it follows a blow or fall on the joint, which it may do, for ataxics are liable to such accidents, an error may be made; but the evolution of the symptoms is so different from those which usually follow local contusions, together with the sudden general swelling of the limb, that a mistake will be soon corrected. The great deformity of the joint at an advanced period of the arthropathy might suggest white swelling, particularly if the patient has been long confined to bed from the principal disease, and here the diagnosis may offer some difficulty. The antecedents of the case must be taken into consideration as the well as the other concomitant symptoms. By recognizing true nature of the affection, Dr. Ball was able to save a patient the loss of a limb, which the surgeon of the hospital had decided on. It is possible to confound ataxic arthropathy of the shoulder-joint with a long-standing, unreduced dislocation; but here there is found under the skin, not the head of the humerus, but more or less complete destruction of the head of the bone.

I can only now allude to the possible *coexistence of locomotor ataxy* and *progressive muscular atrophy.* Cruveilhier, Virchow, Dumenil, and Lockhart Clarke have published cases where the two affections were conjoined. It seems to be of rare occurrence, but, as Clarke remarks, when the two diseases are coincident, and the muscular atrophy is extensive, the proper characters of locomotor ataxy may be so modified or obscured as to be either entirely overlooked, or considered as referrible to the former disease. On the other hand, there are certain cases of muscular atrophy that at an early stage of the disease might be mistaken for locomotor ataxy; as when wasting of the muscles affects the lower extremities, and though at first scarcely perceptible to the eye, it may yet be sufficient to produce unsteadiness of gait. The researches of Valentiner, Luys, Dumenil, of Rouen, Schuppel, Clarke, Hayem, and Charcot seem to connect progressive muscular atrophy with a peculiar alteration of the cells of the gray substance of the anterior cornua of the cord. If this be so, it is easy to understand that the morbid processus which determines these lesions, originally limited to the gray substance, or, more exactly speaking, to certain elements of this substance, may by gradual encroachment reach the white columns; or, conversely, that the morbid change in the white columns, when primarily affected, may extend to the central gray substance. Now, in the former case, the symptoms of locomotor ataxy would come to be added to the pre-existing phenomena of progressive muscular atrophy; whilst in the second, the amyotrophy, on the contrary, would only appear consecutively as a complication.

Each form of *spinal sclerosis* shows a pretty constant preference for given districts of the cord, yet occasionally the several varieties are found commingled. Cruveilhier published, nearly thirty years ago, a case of multilocular sclerosis of the cord (sclerose en plaques disseminees of Charcot), associated with the special structural alterations of the posterior columns proper to locomotor ataxy, under the caption of "Incomplete paraplegia of motion and sensation, with chronic St. Vitus' dance; extensive gray degeneration of the middle and posterior columns of the spinal cord." (*Anat. Pathol.*, Liv. xxxii., p. 19). In an article of Friedreich on "Atrophic degeneration of the posterior columns of the spinal cord," in Virchow's Archives, 1863, there are two instructive cases recorded, showing the coexistence of the two disorders; and we are indebted to Dr. Bourneville for another observation in the service of Dr. Charcot, at the Saltpetriere hospital. (*Nouvelle Etude sur quelques points de la Sclerose en plaques disseminees.* Paris, 1869, p. 205).

Let us pass on to an examination of the mechanism and *pathogeny* of what is looked upon as the distinctive symptom of locomotor ataxy—the so-called motorial inco-ordination, which has its expression in the characteristic helter-skelter gait. By some it is regarded as depending on anæsthesia, cutaneous and deep-seated; others refer it to impaired reflex action; a few believe it due to a loss of the muscular sense of Bell; whilst Dr. Duchenne, of Boulogne, assumes the existence of a special faculty of muscular co-ordination, and is still in search of the physiological unit in some district of the brain (*Physiologie des Mouvements Musculaires.* Paris, 1867, p. 791). When we study the minute anatomy of the spinal cord, which we owe to the unwearying investigations of Dr. Lockhart Clarke, and then look at the anatomical characters of locomotor ataxy, it would seem that there is no need to imagine any special sense or psychical entity to comprehend the nature of the motorial troubles.

Before Dr. Clarke began his researches, it was held that the posterior roots of the spinal nerves are attached exclusively to the lateral columns of the cord; he has shown that they are attached immediately to the posterior columns, and not at all to the lateral. This is an anatomical fact of immense import to the pathogeny of locomotor ataxy.

[The course of each of the three bundles of posterior root-fibres through the posterior columns and gray substance, and the passage of some of them into the anterior columns, were explained and illustrated by photographs after Clarke's drawings. The structural changes in the posterior columns, nerve-roots, and cornua, which are constant in locomotor ataxy, and constitute its anatomical characters, were next described, and photographs, after Clarke, Charcot, Vulpian, and Cornil, showing the appearance of these degenerations, and their histology, were exhibited.]

From his investigations Dr. Clarke inferred (1853), that the posterior white columns cannot be the only channel for the transmission of sensory impressions; and this opinion was confirmed by the experiments of Dr. Brown-Séquard made two years after (1855). The amount of damage always present in the posterior columns and posterior nerve-roots must necessarily interfere with their functions, and as a consequence the correlation of the nervo-muscular systems essential to the accuracy and harmony of certain motory acts will be more or less disturbed. Disturbed sensorial susceptibility is a natural outcome from the lesions of the cord; and is found to happen, and constantly too in some degree, from the very outset. Trustworthy observers, as Leyden, Allbutt, etc., affirm it, and such is my own experience, as already stated. The fact of early or constant anæsthesia is denied by many, and even some of those who admit its presence will not allow that it can be the cause of the motorial troubles, and call on physiological experiments and clinical facts to uphold their opinion. It is undoubtedly true that anæsthesia, in the strict sense of the word, may not be manifest in all stages of some cases of locomotor ataxy, and particularly in the initial stage, though motorial derangement may have already begun; for the sensorial modification is more properly described by the term *dysœsthesia*, or still more correctly *bradyœsthesia* (slow). The impression of the peripheral stimulus may reach the receptive surface intact, and be there appreciated in its entirety, but there is delay in the transmission, and hence delay in the answer. Several seconds may elapse before a skin irritation, as pricking or pinching the skin, or an electric excitation is perceived. In one of Lockhart Clarke's cases, three or four minutes elapsed, and in another there was an interval of twenty minutes before the patient, without being asked, complained of pain from the thrust of a needle (*St. George's Hospital Reports*, vol. i., p. 77, 1866).

I have known instances where the galvanic current was not felt for from two to five seconds.* I have seen ataxics, who could not pick up a pin or a match at once, or handle a small object, be able to do so after resting the pulps of the fingers on the article for a moment or so, until in fact the impression

*One of the hypotheses offered in explanation of this tardy transmission of peripheral excitations attributes the delay to the necessity of their having to pass exclusively through the gray substance of the cord, the posterior columns being more or less damaged; whilst otherwise they would take the more rapid route of the posterior fasciculi. There is an interesting observation of Dr. Vulpian going to sustain this view. In a typical case of locomotor ataxy, the patient, from previous large bilateral hæmorrhages into the cerebrum, was pretty much in the state of an animal in which both hemispheres have been artificially injured. Spinal excitability was increased, and reflex action, unfettered by any brain influence, could manifest itself to its fullest extent. If the skin of the lower part of either leg were sharply pinched, there was almost instantly the evidence of weak reflex action, and the leg was slightly flexed; but after the lapse of a few seconds, a second movement, stronger and of greater extent, of flexion occurred, and lasted four or five seconds. The patient was speechless and semi-conscious, but there was no sign from the expression of the face that the irritation was felt. These second movements were more marked on the side most recently paralyzed.

had been conducted to the perceptive center. So far, indeed, from anæsthesia proper being present in all cases, there may be hyperæsthesia, and the impression when felt be exaggerated both in intensity and duration. If some degree of dysæsthesia is not found in locomotor ataxy, we may suspect that it has not been intelligently looked for. The adjustment of an æsthesiometer or the compasses, and questioning the patient and receiving his answer, give time for the transmission of the impression in most cases, and in this way the observer is misled.*

The recognition of a derangement of tactile sensibility does not, however, prove that it is the sole factor in the production of muscular disharmony. It is an essential one, but is probably only one of several, which together concur in producing the effect known as motorial inco-ordination, which has come to be regarded as the type-sign of locomotor ataxy. But it may be asked whether the characteristic objective symptom — the hitchy precipitate gait — is really an expression of muscular inco-ordination simply, as is universally set forth, or whether it be not in fact a complex phenomenon. In the beginning there is only unsteadiness and uncertainty in the movements, shown in the ready loss of balance, the trunk oscillations, clumsy, staggering walk, and often fumbling fingers. There is lessened ability to guide the movements. All this is seen in many other affections, and is familiar to us as an effect of alcoholic intoxication. But after a while the quality of the walk is changed; it becomes jerky and spasmodic, and we have the peculiar gait which is proper to locomotor ataxy. The defect of prompt muscular adjustment, noticed in the earlier stages of the disease, may perhaps be properly called inco-ordination, and marks the period when formative irritation has set in; but the changes

*Dr. Brown-Séquard has indicated another source of error in appreciating the degree of tactile sensibility in sclerosis of the posterior columns. He takes as an example a case where the lesion is limited to one cord, and involves the whole dorso-lumbar enlargement, of which he has seen three instances. The ataxy is unilateral and in the corresponding limb. On applying the æsthesiometer both points are correctly felt, as well as pinching, pricking, tickling, &c. The conclusion reached is that there is no modification of sensibility. But according to Dr. Brown-Séquard the very fact that tactile sensibility is apparently natural is sufficient to show that it is really lessened, and that too in a high degree; for sclerosis of the posterior columns, as well as other lesions, is a powerful cause of hyperæsthesia; and if the patient does not feel much more distinctly the various impressions made on the ataxic limb, it is because there is really anæsthesia, and that too in the same parts that are hyperæsthetic. If its sensibility seems natural, the reason is that this faculty has lost all it should have gained, if there had not been a cause of anæsthesia coexisting with the cause of hyperæsthesia. It is wrong, therefore, to say that sensibility is natural when it only seems to be so, and we know that it should be increased. (Archives de Physiologie, &c., t. ii., p. 761, 1869.)

in the spinal cord are yet molecular only, or if structurally palpable, are so in a slight degree. The result of the lesion of the cord is a delay in the transmission of the peripheral incitations to the receptive surfaces—dysæsthesia; hence the want of prompt co-operation between the regulating centers and the motor instruments. The balance of relations between the recipio-motor and the dirigo-motor systems, to borrow Mr. Herbert Spencer's terms, which insure perfectness in complete actions, becomes troubled. When later the posterior columns and posterior nerve-roots are extensively and seriously damaged, new perturbations of spinal mechanism happen; the irradiations* are fitful, wild, and more or less uncontrollable; centripetal impressions and centrifugal impulses travel vaguely, the muscles are in mutiny, and the type-gait is established, and sometimes the extravagances of spinal epilepsy are roused. Still later, when the disorganization of the posterior columns and nerve-roots is complete or nearly so, there is true paraplegia, and the unfortunate sufferer becomes bed-ridden.

Although the progressive structural changes in the spinal cord and nerve-roots may account, in a general way and in accordance with physiological observations, for the characteristic gait and other motorial excesses in ataxics, the precise mechanism of these phenomena has been variously explained, and is still a subject of speculation. Time will not allow me to discuss any of these theories, but I cannot help alluding to the ingenious suggestions of Dr. Lockhart Clarke respecting the influence of the loss, more or less partial, of muscular tonicity, a physiological state dependent on the integrity of reflex action.† This normal tension or tone is defined as that moderate but constant state of contraction that keeps antagonist muscles, or those variously opposed to each other, in equilibrium. In all complicated voluntary movements, a constantly varying number of muscles are balanced against each other in contractions. If some of the muscles of the group employed have lost their tone, they will not properly respond to the voluntary stimulus in the execution of a given movement, and will fail to do properly their part in balancing the effects of the other muscles of the group, whose tension is whole. In

*The phenomena of spinal irradiations consist in the propagation of the motorial incitation to those nervous elements which have not directly received it. If from some cause the excitability of the elements is increased, the irradiated excitation will be more intense and more extended. This is one of the explanations that can be given to account for the untimely contraction of the antagonist muscles. (Jaccoud, Traite de Path. Int., t. i., p. 336.)

† British Medical Journal, vol. ii., 1869, p. 344.

proportion, therefore, to the amount of tension lost by any muscle or set of muscles, there must necessarily result a corresponding degree of disorderly movement. The morbid condition of the cord which would cause this lessening or loss of muscular tone, which is subordinate to a sort of reflex action, actually exists, and it is quite probable that the impairment of this property of the muscles may prove one factor in the production of the peculiar gait.

Whatever may be the value of these theories towards the solution of the complex problem of the motorial troubles in locomotor ataxy, they do not help us to a right understanding of the intimate nature of the affection. The genetic cause is still to be sought. The connection between the disorders of the cranial nerves, so frequent at the outset, and later spinal symptoms, with their relation to structural changes in the cord situally remote, without any necessary implication of the intervening nerve-tissue, has not been made out. These phenomena are certainly not accidentally coincident; they are plainly parts of the same morbid processus, whose uniting link is yet wanting. May it not be found with a better knowledge of the physiology and pathology of the sympathetic nerve? The premature senile decay of some ataxics, compared with the persistent, vigorous general nutrition of others, is note-worthy, and merits investigation.

[Dr. C., in reply to an inquiry, further said: The *prognosis* is not so gloomy as was once thought. The disease is sometimes arrested. In many there is more or less amelioration of the symptoms; and in a few cases recovery has happened. There is liability to error in estimating the value of any particular mode of treatment, on account of the natural pauses in its progress. The several methods of treatment recommended have been too haphazard and specific; single remedies have been blindly tried and submitted to statistical test. May not our therapeutics gain something by a study of the patho-anatomy and patho-physiology of the affection? It would seem that the chances of their success are in proportion to the ability to detect the early morbid errors. Even in the first stages, it is probable more can be done by general than by local measures to arrest its course. Setons, issues, cauteries to the spine, would all seem to be harmful; proper food and clothing very important. Whilst faradisation appears, as a rule, to be injurious, the constant galvanic current, on the other hand, has given some remarkable results. Cases of considerable improvement, and even of recovery, from its use, have been reported by Remak, Frommhold, Cyon, Benedikt, Onimus, and others. My own more limited experience is favorable to it. At my suggestion the Calabar bean (physostigma ven.) has been tried in one case, and with apparent benefit to the motorial troubles. The local pains may be ameliorated by cannabis indica, subcutaneous injections of atropia and morphia. When localized and permanent, they are best relieved by chloroform applied directly to the part.]—*Medical Record.*

